# Combined therapy with RAD001 e BEZ235 overcomes resistance of PET immortalized cell lines to mTOR inhibition

**DOI:** 10.18632/oncotarget.2111

**Published:** 2014-06-18

**Authors:** Ilaria Passacantilli, Gabriele Capurso, Livia Archibugi, Sara Calabretta, Sara Caldarola, Fabrizio Loreni, Gianfranco Delle Fave, Claudio Sette

**Affiliations:** ^1^ Department of Biomedicine and Prevention, University of Rome Tor Vergata, Rome, Italy; ^2^ Digestive & Liver Disease Unit, University of La Sapienza, Rome, Italy; ^3^ Department of Biology, University of Rome Tor Vergata, Rome, Italy; ^4^ Laboratory of Neuroembryology, Fondazione Santa Lucia, Rome, Italy

**Keywords:** PETs, mTORC1 chemical inhibitors, RAD001, BEZ235, adaptation to chronic treatment, combined treatment

## Abstract

Pancreatic endocrine tumors (PETs) are characterised by an indolent behaviour in terms of tumor growth. However, most patients display metastasis at diagnosis and no cure is currently available. Since the PI3K/AKT/mTOR axis is deregulated in PETs, the mTOR inhibitor RAD001 represents the first line treatment. Nevertheless, some patients do not respond to treatments and most acquire resistance. Inhibition of mTOR leads to feedback re-activation of PI3K activity, which may promote resistance to RAD001. Thus, PI3K represents a novel potential target for PETs. We tested the impact of three novel PI3K inhibitors (BEZ235, BKM120 and BYL719) on proliferation of PET cells that are responsive (BON-1) or unresponsive (QGP-1) to RAD001. BEZ235 was the most efficient in inhibiting proliferation in PET cells. Furthermore, combined treatment with BEZ235 and RAD001 exhibited synergic effects and was also effective in BON-1 that acquired resistance to RAD001 (BON-1 RR). Analysis of PI3K/AKT/mTOR pathway showed that RAD001 and BEZ235 only partially inhibited mTOR-dependent phosphorylation of 4EBP1. By contrast, combined therapy with the two inhibitors strongly inhibited phosphorylation of 4EBP1, assembly of the translational initiation complex and protein synthesis. Thus, combined treatment with BEZ235 may represent suitable therapy to counteract primary and acquired resistance to RAD001 in PETs.

## INTRODUCTION

Pancreatic endocrine tumors (PETs) are rare neoplasms that represent 1-2% of all pancreatic cancers [1, 2]. They are heterogeneous in terms of clinical presentation, histological features, tumor grading and staging at time of diagnosis [3, 4]. Despite PETs are considered to have an “indolent” behaviour, most patients display metastases at the time of diagnosis, being not eligible for surgery [3]. Furthermore, as PETs are characterised by a low proliferation rate, chemotherapy is chosen as first line treatment only for a subgroup of patients with more aggressive features, whereas treatment of the majority of PETs has generally employed somatostatin analogues [4]. Nevertheless, these therapeutic approaches offer limited clinical benefits for patients.

In the past few years, this therapeutic scenario has dramatically changed. Two novel targeted therapies, with the selective inhibitor for the serine-threonine kinase mTOR (Everolimus or RAD001) and the multi-target tyrosine-kinase inhibitor sunitinib, have been approved for advanced progressive pancreatic neuroendocrine tumors (pNETs) [5, 6]. In particular, the rationale for the use of RAD001 in PETs is sustained by solid preclinical data with human samples and in vitro models that highlighted the relevance of the PI3K-mTOR pathway in PETs [7-10]. For instance, low expression levels of negative regulators of mTOR, such as PTEN and the TSC1/2 complex, are associated with worse prognosis in PET patients [7]. Moreover, a number of studies have confirmed the efficacy of RAD001 in models of neuroendocrine tumours in vitro [11, 12]. However, despite the strong rationale and the specific mechanism of action of RAD001, not all patients respond to the treatment. Indeed, one third of patients display primary insensitivity [13], whereas others initially experience disease stabilization but they eventually develop resistance to the drug and undergo disease progression [5].

The PI3K/AKT/mTOR axis is involved in the regulation of cell survival, proliferation and motility [14]. mTOR is assembled in two main complexes named mTORC1, which regulates mRNA translation and protein synthesis in response to nutrients [15], and mTORC2, which is mainly involved in cytoskeleton remodelling and cell survival [16]. Notably, RAD001 specifically targets the mTORC1 complex whereas mTORC2 is insensitive to it, thus leaving some mTOR functions unaltered upon treatment [15, 16]. Importantly, mTORC1 participates to a negative feedback that keeps the activity of PI3K under tight control [17]. As a consequence, mTORC1 inhibition can lead to activation of PI3K and of the pro-survival kinase AKT [18]. Thus, novel therapeutic strategies that avoid instauration of feedback activation of the PI3K/AKT axis might be beneficial in long-term treatments of PET patients with mTORC1 inhibitors.

In this work, we aimed at evaluating the response of PET cell lines to three novel PI3K inhibitors, the dual PI3K-mTOR inhibitor BEZ235 [19], the pan-PI3K inhibitor BKM120 [20] and the PI3Kα inhibitor BYL719 [21]. BEZ235 was the most efficient among the PI3K inhibitors in limiting PET cell growth. Notably, although BEZ235 alone did not provide an advantage with respect to RAD001, combined treatment with these inhibitors could overcome the resistance of PET cells to RAD001 and it significantly lowered the dose required to exert anti-proliferative effects. The synergic effect relied on more efficient inhibition of 4EBP1 phosphorylation, consequent impairment of the assembly of the translation initiation complex eIF4F and strong inhibition of protein synthesis. Thus, our results demonstrate *in vitro* the efficacy of combined treatment with RAD001 and BEZ235 in PET cells, providing the basis for studies using *in vivo* models of PET.

## RESULTS

### Establishment of a PET cell model of acquired resistance to RAD001

**Figure 1:**
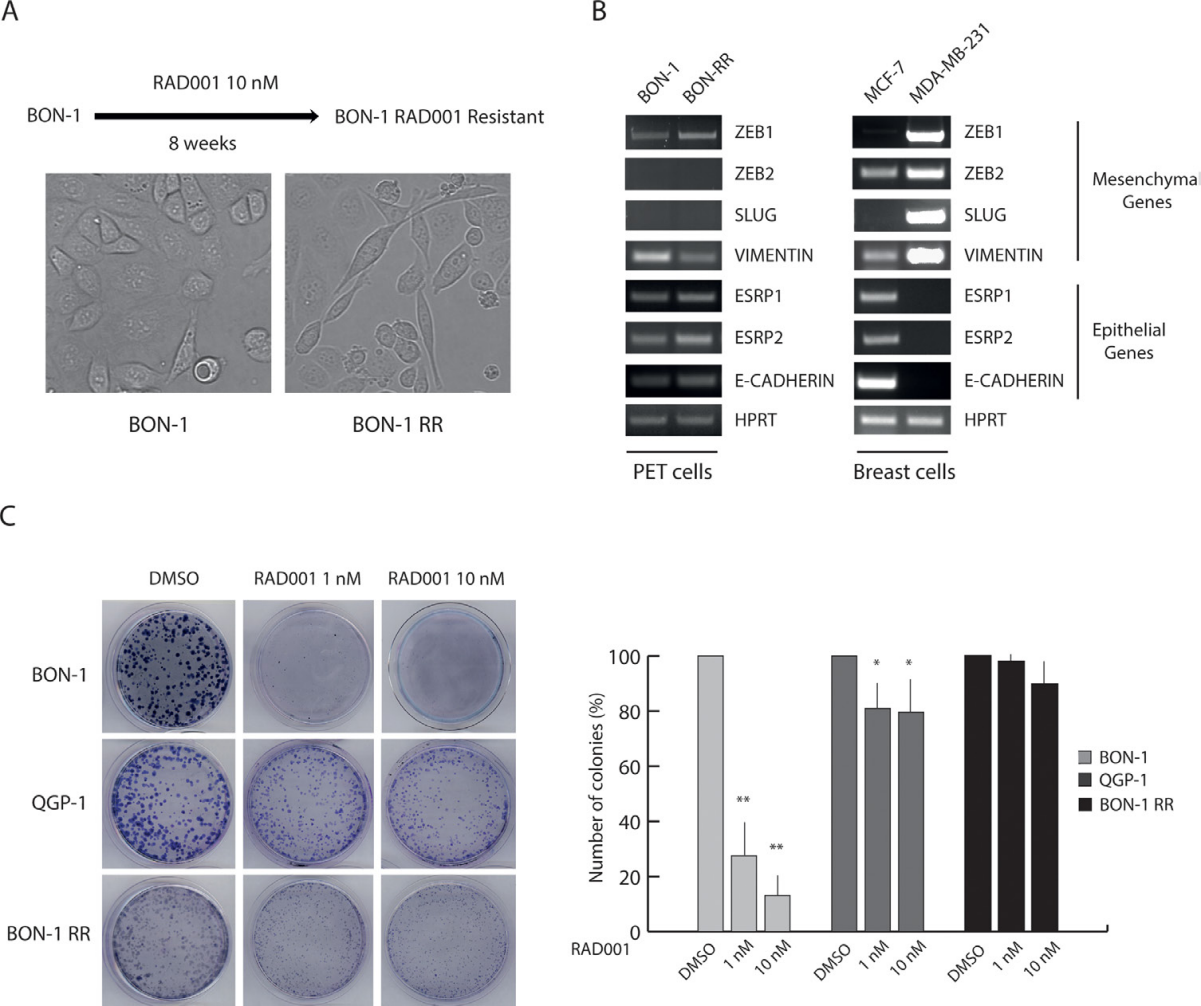
Chronic treatment selects RAD001-resistant BON-1 cells (A) Scheme of the protocol used to select a RAD001-Resistant BON-1 cell line (BON-1 RR). Representative images of parental and RAD001-resistant BON-1 cells. BON-1-RR show a more elongated shape and fewer cell-cell contacts with respect to the morphology of parental cell (40X magnification). (B) RT-PCR analysis of the expression of mesenchymal and epithelial genes in BON-1 and BON-1 RR cells. MCF-7 and MDA-MB-231 breast cancer cells were used as positive control of epithelial and mesenchymal phenotype, respectively. (C) Representative images of colony formation assay performed with BON-1, QGP-1 and BON-1 RR treated with 1 or 10 nM RAD001. Histograms represent the percentage of inhibition of colony formation in comparison to control cells from three experiments (mean ± s.d.). Statistical analysis was performed by the paired Student's t-test; ^*^ P ≤ 0.05, ^**^ P ≤ 0.01.

Clinical data indicate that a subset of PET patients respond to RAD001 treatment with tumor regression or stabilization, whereas others display primary resistance. In addition, the majority of patients that initially respond to the treatment then develop secondary resistance within 1 year [13]. We aimed at developing cell models representing these clinical situations to test the effect of three novel PI3K inhibitors in PETs. The PET cell lines BON-1 and QGP-1 exhibit a different sensitivity to RAD001 in terms of proliferation, with BON-1 cells being highly sensitive to the inhibitor and QGP-1 rather resistant [7, 10]. To determine whether RAD001-sensitive cells could acquire resistance to the drug, we treated BON-1 cells with RAD001 for 8 consecutive weeks. RAD001 (10 nM) was supplied every 48 hours together with fresh medium (Figure 1A). Treatment with RAD001 almost completely blocked proliferation of BON-1 cells in the first week (Supplementary Figure 1A). However, after 10-15 days of treatment cells started to grow slowly and by the end of the treatment they exhibited a proliferation rate in the presence of RAD001 that was comparable to that of parental BON-1 cells in the absence of the drug (Supplementary Figure 1B). These cells, which we named BON-1 RR (RAD001 Resistant) for their acquired phenotype, displayed a more elongated shape and fewer cell-cell contacts with respect to the morphology of parental cells (Figure 1A). Although changes in elongated shape are often a hallmark of epithelial-to-mesenchymal transition in cancer cells, as exemplified by the MCF-7 and MDA-MB-231 breast cancer cells (Figure 1B), we found that this is not the case for BON-1 cells. Indeed, parental BON-1 cells express mixed markers of both epithelial and mesenchymal phenotype and their expression levels are not significantly changed in BON-1 RR cells (Figure 1B).

To validate the differential sensitivity of PET cell lines to RAD001, we performed colony formation assays, which measure the ability of cells seeded at clonal dilutions to form new colonies [22]. As expected, parental BON-1 cells were highly sensitive to RAD001, with approximately 75-90% inhibition of colony formation at 1-10 nM concentrations (Figure 1C). QGP-1 cells were substantially resistant to the drug, which caused a 20-35% reduction in number of colonies (Figure 1C). Strikingly, BON-1 RR cells were strongly resistant to RAD001, with approximately 10% reduction in colony formation at the highest dose (Figure 1C). These results suggest that PET cells that are sensitive to mTORC1 inhibition can develop resistance to RAD001 treatment, similarly to what observed in patients [5, 13].

### PI3K inhibitors display different efficacy in the inhibition of PET cell growth

In various cancer cell lines, inhibition of mTORC1 activity causes a feedback activation of PI3K and phosphorylation of AKT, resulting in a pro-survival response [18]. To test whether such feedback control is also active in PET cells, we treated BON-1 and QGP1 cells with different doses of RAD001. Notably, RAD001 induced sustained (4-24 hours) phosphorylation of AKT in Thr 308 and Ser 473 in both PET cell lines (Supplementary Figure 2). These results suggest that the prosurvival PI3K/AKT pathway is activated in both RAD001-sensitive and -resistant PET cells.

**Figure 2:**
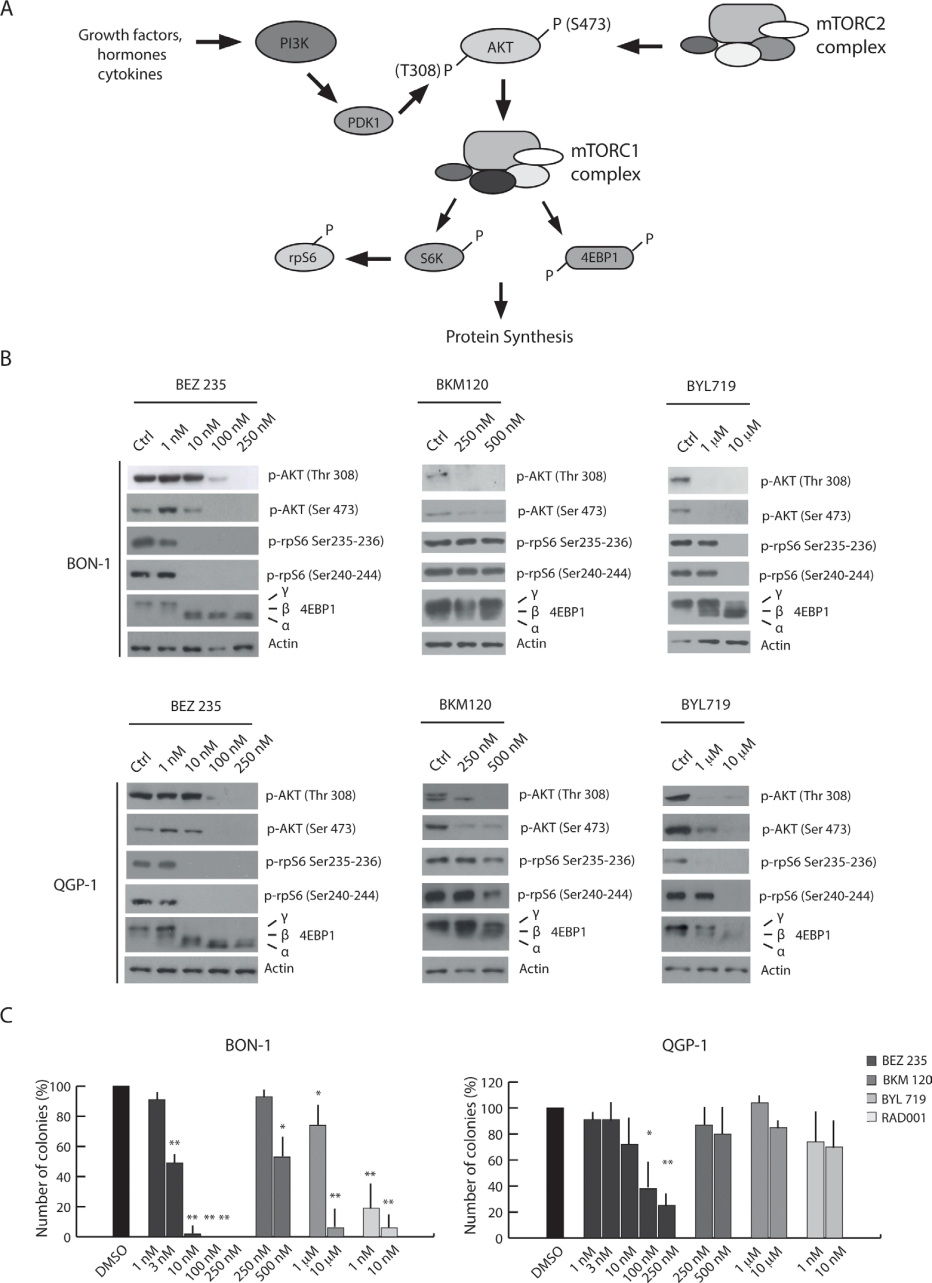
PI3K inhibitors display different efficacy in the inhibition of PET cell growth (A) Schematic representation of the PI3K/Akt/mTOR pathway. (B) Western blot analysis of 4EBP1, p- AKT Ser 473, p-AKT Thr 308, p-rpS6 Ser 240-244 and p-rpS6 Ser 235-236 in BON-1 and QGP-1 treated with the PI3K inhibitors BEZ235, BKM120 and BYL719 for 4 hours. Actin was used as loading control. (C) Colony formation assays performed in BON-1 and QGP-1 treated with the PI3K inhibitors and RAD001. Histograms show the percentage of inhibition of colony formation in comparison to control cells from three experiments (mean ± s.d.). Statistical analysis was performed by the paired Student's t-test; ^*^ P ≤ 0.05, ^**^ P ≤ 0.01.

BEZ235 is a dual inhibitor that inhibits the catalytic activity of mTOR and of all class I PI3K isoforms by targeting their ATP binding site [19]. BKM120 acts on all class I PI3K isoforms [20], whereas BYL719 specifically inhibits the activity of the p110α catalytic isoform [21]. To evaluate the activity of these compounds in PET cells, we initially tested the minimal dose of each drug required to inhibit AKT phosphorylation and mTORC1 activity in BON-1 and QGP-1 cells (Supplementary Figure 3). Phosphorylation of AKT in Thr 308, which is mediated by PDK1, was evaluated as marker of PI3K activity, phosphorylation of AKT in Ser 473 was evaluated as target of mTORC2 activity, whereas phosphorylation of rpS6 and of 4EBP1 were evaluated as downstream targets of mTORC1 (see Figure 2A for pathway representation).

In both cell lines, BEZ235 inhibited mTORC1 activity at 10 nM, as indicated by reduced phosphorylation of rpS6 and the shift to faster electrophoretic mobility of 4EBP1 (α isoform in Figure 2B). However, at 100-250 nM BEZ235 also impaired PI3K and mTORC2 activities, as shown by reduced phosphorylation of AKT in Thr 308 and Ser 473, respectively (Figure 2B). By contrast, the other two drugs were less effective in inhibiting these pathways and required much higher dosage (Supplementary Figure 3A,B). BKM120 inhibited PI3K (AKT Ser308) and partially mTORC1/2 activities at 250-500 nM, whereas BYL719 was active at concentrations in the micromolar range (1-10 μM) (Figure 2B and Supplementary Figure 3A,B). Specific inhibition of mTORC1 activity by BEZ235 was also tested by monitoring phosphorylation of mTOR in Ser 2448, a substrate of the ribosomal S6 kinase (23) that is activated by the mTORC1 complex (15). As expected, we found that increasing doses of BEZ235 reduced mTOR phosphorylation, whereas the PI3K-specific inhibitors were ineffective (BKM120) or exerted a partial effect only at high doses in the RAD001-sensitive BON-1 cells 10 μM BYL719) (Supplementary Figure 3C,D).

**Figure 3:**
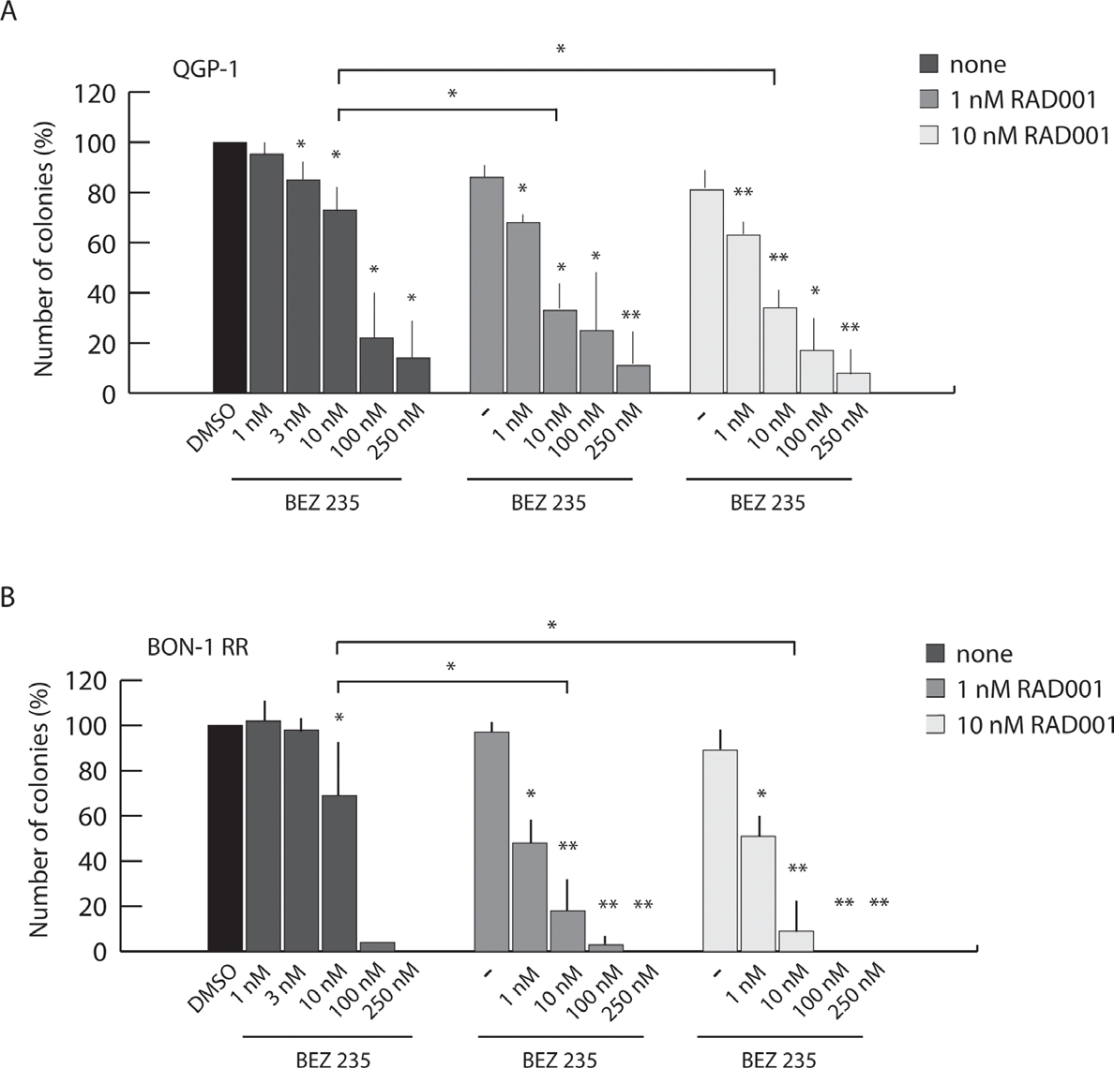
Combined treatment with BEZ235 and RAD001 overcomes resistance of PET cells to RAD001 Colony assay performed in QGP-1 (A) and BON1-RR (B) treated with BEZ235, RAD001 or both inhibitors as indicated. Histograms show the percentage of inhibition of colony formation in comparison to control cells from three experiments (mean ± s.d.). Statistical analysis was performed by the paired Student's t-test; ^*^ P ≤ 0.05, ^**^ P ≤ 0.01.

In order to evaluate the effect of the PI3K inhibitors on PET cell proliferation and survival, we performed colony formation assays. In BON-1 cells, BEZ235 did not confer an advantage with respect to RAD001 and suppressed colony formation at 3-10 nM concentration (Figure 2C), a dose at which this inhibitor affected mTORC1 activity but not PI3K activity (Figure 2B). By contrast, in QGP-1 cells, which are rather resistant to RAD001 and to doses of BEZ235 that inhibit only mTORC1, increasing doses of BEZ235 significantly inhibited colony formation and growth with respect to the effect of RAD001 (Figure 2C). This result might indicate that inhibition of PI3K activity overcomes RAD001 resistance in PET cell lines. However, BKM120 and BYL719 did not suppress QGP-1 colony formation even at doses that efficiently inhibited PI3K activity, whereas they were more efficacious in BON-1 cells (Figure 2C). Thus, these observations suggest that the concomitant inhibition of mTORC1 and PI3K/mTORC2 exerted by high doses of BEZ235 is beneficial to limit growth of RAD001-resistant PET cells.

### Combined treatment with BEZ235 and RAD001 overcomes resistance of PET cells to RAD001

To test whether combined treatment with BEZ235 and RAD001 provides an advantage with respect to single treatment with each drug, we evaluated their effect in PET cells that are resistant to RAD001 alone (QGP-1 and BON-1 RR cells) by colony formation assays. As shown above (Figure 2C), QGP-1 cells were rather resistant to BEZ235 at concentrations (1-10 nM) that selectively inhibited mTORC1, whereas their growth was strongly inhibited at concentrations (100-250 nM) that also suppress PI3K and mTORC2 activity (Figure 3A). A similar result was observed for BON-1-RR cells (Figure 3B). However, when low doses of BEZ235 (1-10 nM) were administered in combination with 1 nM RAD001, growth was significantly inhibited in both QGP-1 (~25-65% inhibition, Figure 3A) and BON-1-RR (~50-80% inhibition, Figure 3B) cells. Importantly, increasing the dose of RAD001 to 10 nM did not provide a significant amelioration of the effect of BEZ235 (Figure 3A,B). Direct measurement of cell proliferation and cell death indicated that the combined treatment mainly affected proliferation of PET cells (Supplementary Figure 4A,B). Notably, co-treatment of cells with RAD001 and BKM120, which inhibits only PI3K, did not exert a synergic effect on PET cell proliferation (Supplementary Figure 5). These data indicate that combined treatment of PET cells with RAD001 and BEZ235 is more effective with respect to the action of each drug alone. Furthermore, the combined treatment allows lowering tenfold the minimal concentration of both drugs required to exert significant inhibition of PET cell growth.

### Combined treatment with BEZ235 and RAD001 efficiently suppresses phosphorylation of AKT and 4EBP1 in RAD001-resistant PET cells

**Figure 4:**
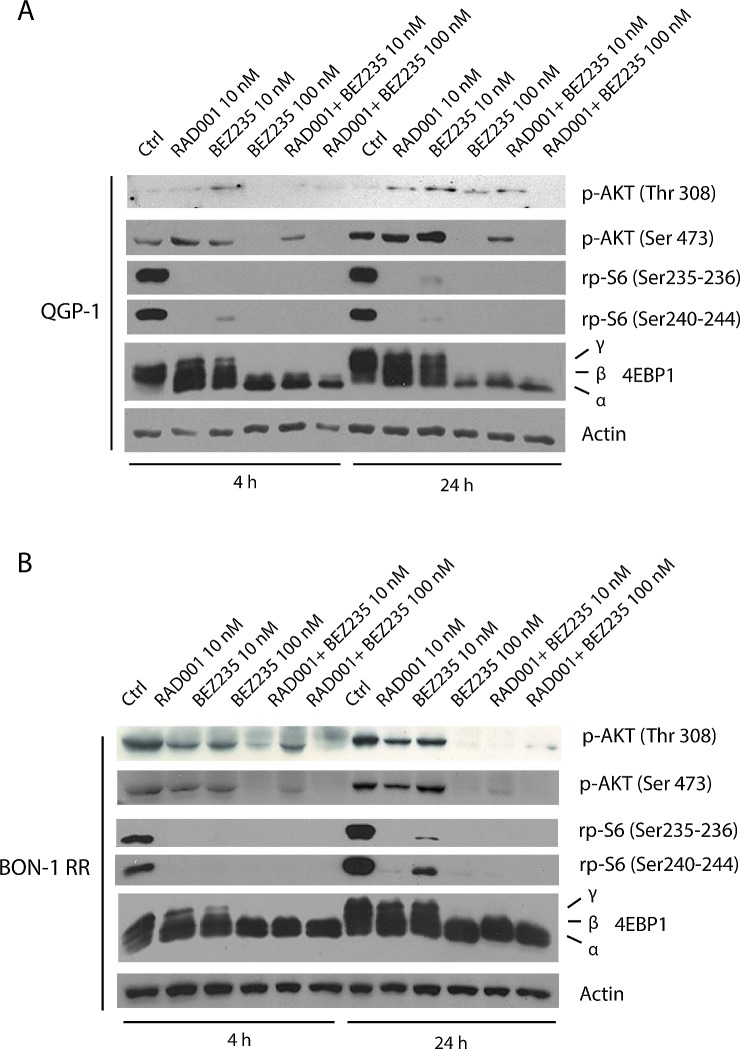
Combined treatment with BEZ235 and RAD001 efficiently suppresses phosphorylation of AKT and 4EBP1 in RAD001-resistant PET cells 4EBP1, p-AKT Ser 473, p-AKT Thr 308, p-rpS6 Ser 240-244 and p-rpS6 Ser 235-236 in QGP-1 (A) and BON-1 RR (B) treated with the BEZ235 (indicated dose), RAD001 (10 nM) or both inhibitors. Actin was used as loading control.

To investigate the molecular mechanism(s) underlying the synergic effect of BEZ235 and RAD001 in PET cells, we performed Western blot analyses of relevant targets of the PI3K and mTORC1 pathway. We found that in both QGP-1 (Figure 4A) and BON-1 RR (Figure 4B), treatment with 10 nM RAD001 or 10 nM BEZ235 alone did not completely block mTORC1 activity, as indicated by the substantial amount of high molecular weight forms (β and γ isoforms) of its direct substrate 4EBP1. In addition, under these conditions AKT remained phosphorylated in Thr 308 and Ser 473. By contrast, when RAD001 and BEZ235 were administered together (10 nM each), 4EBP1 phosphorylation was completely suppressed, whereas AKT phosphorylation was attenuated in QGP-1 cells (Figure 4A) and abolished in BON-1-RR cells (Figure 4B). Similarly to what observed with the colony formation assay, concomitant inhibition of PI3K/mTORC2 and mTORC1 pathways could be obtained by raising the concentration of BEZ235 alone to 100 nM. These results suggest that the synergism between RAD001 and BEZ235 is due to more efficient inhibition of both the PI3K/mTORC2 and the mTORC1 pathways elicited by the combined treatment (see Discussion).

### Combined treatment with BEZ235 and RAD001 is required to inhibit protein synthesis in RAD001-resistant PET cells

**Figure 5:**
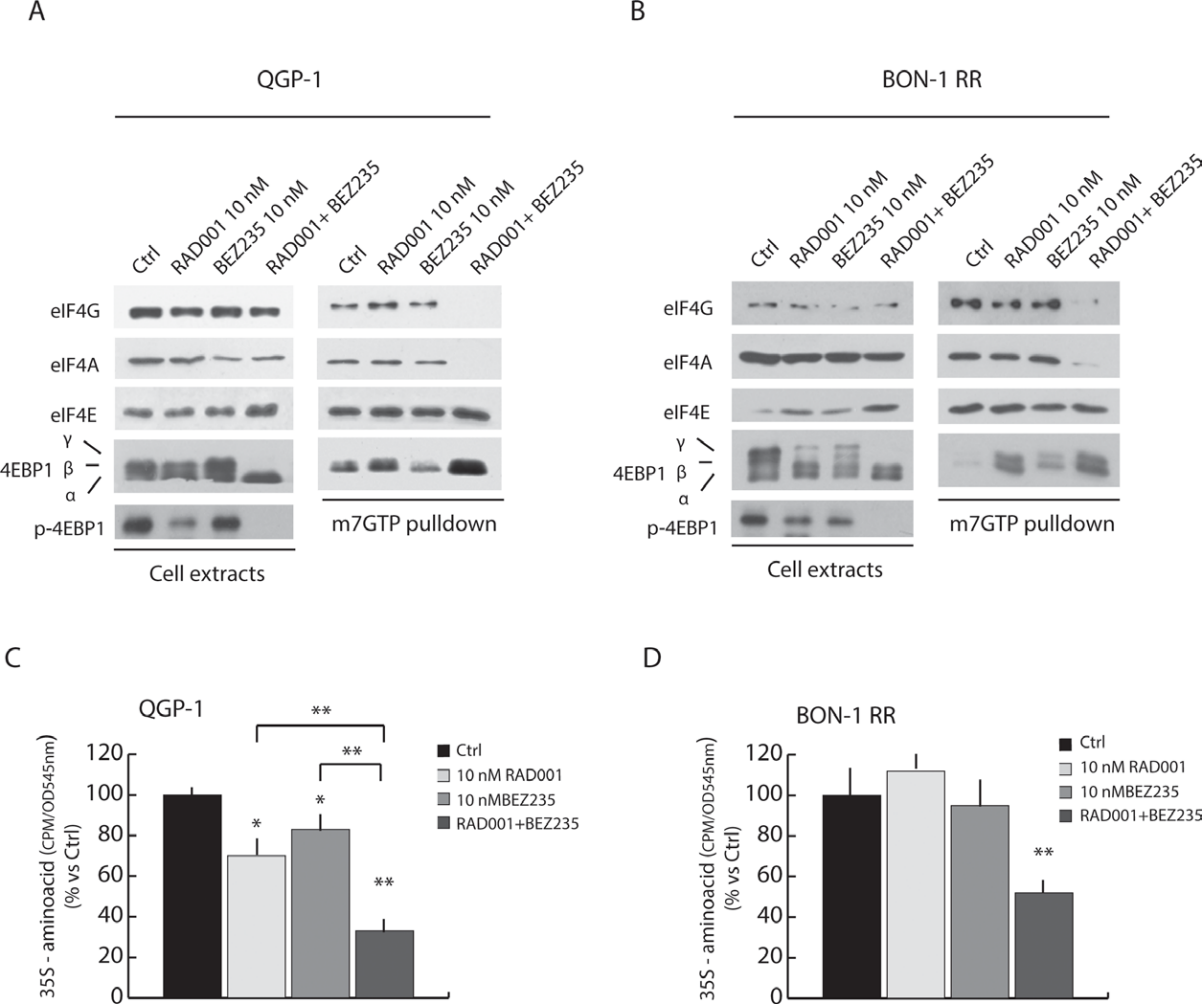
Combined treatment with BEZ235 and RAD001 efficiently suppress assembly of the translation initiation complex eIF4F and protein synthesis in RAD001-resistant PET cells 7-methyl-GTP Sepharose Assay in QGP-1 (A) and BON-1 RR (B) treated with RAD001 (10 nM), BEZ235 (10 nM) or both inhibitors (10 nM each). The proteins absorbed to 7-methyl-GTP-Sepharose beads were analyzed in Western blot with antibody for eIF4G, eIF4A and 4EBP1. (C-D) Protein synthesis was measured by ^35^S-aminoacids incorporation in QGP-1 (C) and BON-1 (D) cell lines. Cells were treated for 72 hours with inhibitors as indicated in the legend. The inhibitors were supplied at T=0 hours and at T= 36 hours together with fresh medium. ^35^S-aminoacid mix was added in the last 30 min of the culture. Results of ^35^S-aminoacid incorporation are expressed as mean ± s.d. of three experiments. Statistical analysis was performed by the paired Student's t-test; ^*^ P ≤ 0.05, ^**^ P ≤ 0.01.

A key function of the mTORC1 complex is the regulation of mRNA translation initiation complex [24]. Phosphorylation of 4EBP1 by mTORC1 inhibits its interaction with the eukaryotic initiation factor eIF4E. This allows recruitment of the scaffold protein eIF4G and the RNA helicase eIF4A by eIF4E to form the active eIF4F translation initiation complex at the 5'-cap of mRNAs and triggers translation initiation [24]. To test whether combined treatment with RAD001 and BEZ235 affected translation initiation, we performed 5'-methyl-cap assays to pull down eIF4E and its associated proteins [25]. In the absence of treatment, eIF4E was associated with substantial amount of eIF4G and eIF4A in both QGP-1 (Figure 5A, right panel) and BON-1-RR (Figure 5B, right panel) cells, indicating efficient assembly of eIF4F. Treatment with RAD001 or BEZ235 alone did not significantly reduce eIF4F formation, even though a partial de-phosphorylation of 4EBP1 was observed in the cell extracts (Figure 5A,B, left panels). By contrast, concomitant treatment with RAD001 and BEZ235 completely suppressed 4EBP1 phosphorylation and promoted its strong association with eIF4E and disassembly of eIF4F, as demonstrated by the strong reduction in eIF4G and eIF4A bound to eIF4E (Figure 5A,B).

The results illustrated above suggest that the effect of combined treatment with RAD001 and BEZ235 on PET cell growth correlates with the inhibition of translation initiation and consequent reduction in protein synthesis. To directly test this hypothesis, we performed metabolic labelling of PET cells with a mix containing ^35^S-labeled aminoacids to measure the effect of RAD001 and BEZ235 on protein synthesis. We found that RAD001 or BEZ235 alone had mild (QGP-1 cells, Figure 5C) or no effect (BON-1 RR cells, Figure 5D) on protein synthesis. By contrast, when the two inhibitors were administered in combination, protein synthesis was strongly reduced in both QGP-1 cells (70%, Figure 5C) and BON-1 RR cells (55%, Figure 5D), confirming the results on the effect on eIF4F assembly. Notably, although in QGP-1 cells each inhibitor could slightly reduce protein synthesis, combined treatment exerted a significantly stronger effect than each single agent (brackets in Figure 5C), similarly to what observed on cell growth and eIF4F assembly.

Collectively, these observations strongly indicate that combined treatment with RAD001 and BEZ235 exerts a synergic effect on PET cell growth through enhanced inhibition of the PI3K/AKT/mTOR axis, impairment of eIF4F complex assembly and consequent reduction of protein synthesis.

## DISCUSSION

Everolimus (RAD001) is currently employed as first line agent for advanced, progressive PETs [5, 13, 26, 27]. The molecular target of RAD001 is the PI3K/AKT/mTORC1 axis, which is often deregulated in PET patients [13, 26] and in other types of human cancers [24, 28]. Unfortunately, however, a relevant number of PET patients show a primary resistance to treatment with RAD001 or acquire a secondary resistance after chronic exposure [13, 26]. Furthermore, clinical trials have demonstrated that RAD001 delays but does not block PET progression [27]. PET cell models showing sensitivity to RAD001 (BON-1) or primary resistance to the drug (QGP-1) have been already described [7, 10]. Herein, we have established a new PET cell model for the study of acquired resistance to RAD001. BON-1 cells were exposed to the drug chronically for 8 weeks, until their growth rate became similar to that of parental cells in the absence of RAD001 (BON-1-RR cells). Direct examination of the sensitivity of BON-1 RR to RAD001 confirmed their increased resistance to mTORC1 inhibition. Thus, we employed BON-1, QGP-1 and BON-1-RR cells as in vitro models of the clinical cases of PET patients to test the efficacy of drugs that target the PI3K/AKT/mTORC1 axis at different levels.

RAD001 is a synthetic analogue (rapalog) of rapamycin, which functions as an allosteric inhibitor of the mTORC1 complex [29]. The sensitivity to rapalogs has been associated to low eIF4E/4EBP1 ratios, whereas increased eIF4E expression and/or decreased 4EBP1 expression confer resistance in cancer cells [30]. However, we found that the eIF4E/4EBP1 ratio was not significantly different in RAD001-sensitive (BON-1) and insensitive (QGP-1 and BON-1 RR) PET cells (Supplementary Figure 6), suggesting that another mechanism is involved.

Adaptation of cancer cells to chronic treatment with rapalogs has been attributed to feedback activation of the PI3K/AKT pathway and to the consequent induction of prosurvival responses in cancer cells [17, 30]. Moreover, for unknown reasons rapalogs efficiently block mTORC1-dependent S6 kinase activation but poorly suppress mTORC1-dependent phosphorylation of 4EBPs [30]. This weakness can be, however, overcome by new generation mTOR inhibitors that directly target the catalytic activity of the kinase [31, 32]. Herein, we aimed at determining whether inhibition of PI3K activity could be beneficial to counteract growth of PET cell lines that are, or become, resistant to rapalogs. Among the inhibitors tested, BEZ235 resulted the most efficient in terms of inhibition of the PI3K/AKT/mTORC1 pathway and cell proliferation. Interestingly, BEZ235 is a dual inhibitor that targets the catalytic activity of PI3K, mTORC1 and mTORC2 [19]. However, we found that BEZ235 apparently inhibits these kinases at different concentrations in PET cells, with mTORC1 activity being suppressed at 1-10 nM while higher doses (100-250 nM) were required to inhibit PI3K and mTORC2. Notably, BON-1 RR and QGP-1, which show resistance to RAD001, do not respond to BEZ235 at doses that impair solely mTORC1 activity, but are sensitive to higher doses at which PI3K and mTORC2 are also blocked. Inhibition of PI3K activity *per se* does not explain the sensitivity of PET cells to high doses of BEZ235, as the other two PI3K inhibitors tested (BKM120 and BYL719) had marginal effects on cell growth despite their ability to suppress PI3K-dependent phosphorylation of AKT. Thus, the higher activity of BEZ235 resides in its ability to concomitantly suppress PI3K and mTOR activity at higher doses.

The clinical use of BEZ235 is currently being evaluated in a variety of solid tumors [33]. A limitation to its employment at high doses might potentially be represented by development of toxicity. On the other hand, the clinical limitation of RAD001 is represented by development of resistance in patients [13, 26, 27]. To determine whether a combined treatment with RAD001 and BEZ235 could circumvent these problems, we exposed PET cells to both drugs. Our study revealed two important findings. First, we found that co-treatment with the two drugs exerted a synergic effect on PET cell proliferation, with up to 80% inhibition obtained with doses of each compound that caused marginal effects when supplied alone. Moreover, we found that the synergic effect of BEZ235 was elicited at doses that efficiently inhibited mTORC1 but not PI3K activity. This effect might be due to the different nature of mTOR inhibition by RAD001 and BEZ235, with the former acting allosterically by binding to FKB12 and the latter directly on the catalytic activity of the kinase [32]. It should also be reminded that although at the 10 nM concentration BEZ235 appears to selectively block mTORC1 in PET cells, recent evidence suggest that extensive crosstalk between the mTORC1 and mTORC2 complexes exist [34] and may enhance the effect of this drug in vivo. Moreover, since 10 nM overcomes the IC50 of BEZ235 on PI3Ks (19), it is also possible that, although we do not detect an inhibition of AKT phosphorylation, PI3K activity might be partially inhibited and this could affect PET cell proliferation. Another possibility is that BEZ235 exerts its synergic effect by inhibiting another kinase beside PI3K and mTOR. However, although we cannot completely rule out this possibility, it remains unlikely, as in vitro studies demonstrated an extreme specificity of BEZ235, with all other kinases tested being inhibited at doses much higher than dose employed in our study [19]. Thus, our results suggest that targeting the mTORC1 complex with allosteric and catalytic inhibitors synergistically impairs PET cell proliferation. In this regard, BEZ235 may present several advantages with respect to other catalytic inhibitors of mTOR [30, 35], because the clinical use of BEZ235 is already in a more advanced stage (see below) and this drug can concomitantly inhibit the PI3K pathway, thus possibly reinforcing growth inhibition.

Previous studies in other cancer cell types indicated that limited or absent inhibition of phosphorylation of 4EBPs was the main cause of resistance to rapalogs and that this resistance could be eliminated by new generation inhibitors targeting the kinase activity of mTOR [30, 35]. However, our results indicate that combined treatment with RAD001 and BEZ235 results in much stronger growth inhibition than either drug alone in PET cells, suggesting that the effect is not simply due to stronger efficacy of catalytic inhibitors with respect to rapalogs. Other studies also suggest that combined treatment with RAD001 and BEZ235 may exert a synergic effect on cell survival and proliferation in other cancer types [36, 37], although the mechanism underlying this effect was not addressed. Here, we found that the anti-proliferative effect of the combined therapy directly correlates with the ability to suppress 4EBP1 phosphorylation and to interfere with the assembly of the translation initiation complex in PET cells. Indeed, our data show that RAD001 and BEZ235 used as single agents only partially reduced phosphorylation of 4EBP1 and interaction of eIF4E with eIF4G and eIF4A, whereas they abolished 4EBP1 phosphorylation and eIF4F assembly when administered together. This effect is biologically relevant, as it was reflected in a much stronger inhibition of protein synthesis in BON-1 RR and QGP-1 cells with respect to cells treated with RAD001 or BEZ235 alone. Thus, concomitant allosteric and catalytic inhibition of mTORC1 is necessary to efficiently circumvent primary and secondary resistance to RAD001 in PET cell lines.

BEZ235 is currently under evaluation in a clinical trial that investigates its use in patients with advanced PETs after failure of RAD001 (ClinicalTrials.gov Identifier:NCT01658436). In this regard, we found that BEZ235 had minor effects on RAD001-resistant PET cells at doses at which it completely suppressed mTORC1-dependent S6K activity. This result suggests that acquired resistance to rapalogs in PET cells cannot be overcome by treatment with catalytic inhibitors of mTORC1. By contrast, increasing the dose of BEZ235 to 100 nM, at which PI3K and mTORC2 activities are also inhibited, strongly suppressed PET cell growth. Thus, BEZ235 might be beneficial as second line agent but it is likely that the high dosage required for cell growth inhibition will cause unwanted responses in patients. Nevertheless, our findings suggest that addition of low doses of RAD001 may strongly sensitize PET cells to BEZ235 and potentially limit the onset of adverse responses in patients, as the combined therapy reduced the dose of each drug required to obtain efficient tumor growth inhibition in vitro. Notably, such strategy is under evaluation in different solid tumors [33], but not in PETs to the best of our knowledge.

In conclusion, our study suggests that combined therapy with RAD001 and BEZ235 might be particularly beneficial to patients that become insensitive to RAD001 treatment and provide the basis to test this hypothesis in a preclinical model of PET *in vivo*.

## MATERIAL AND METHODS

### Inhibitors

RAD001 and the PI3K inhibitors (BEZ235, BKM120 and BYL719) were generously provided by Novartis Oncology (Basel, Swiss). Inhibitors were dissolved in dymethil sulfoxide (DMSO, Sigma- Aldrich), and the stock solutions were diluted to final concentrations in medium.

### Cell cultures, treatments and extracts preparation

BON-1 and QGP-1 cells were cultured as previously described [10, 38]. BON-1 RR (RAD001-Resistant) cells were obtained after chronic treatment with RAD001 for eight weeks. During treatment, 10 nM RAD001 was added to the cell culture every 48 hours.

For western blot analysis, cells were seeded at 70% of confluence. After 24h medium was changed and inhibitors was added to the cultures. After incubation, cells were washed with ice-cold phosphate buffered saline (PBS) and resuspended in lysis buffer (100mM NaCl, 15mM MgCl2, 30mM Tris-HCl pH 7.5, 1mM dithiothreitol, 2mM Na-ortovanadate, Protease-Inhibitor Cocktail (Sigma-Aldrich) and 1% Triton X-100). Cells were incubated on ice for 10 minutes and protein extracts were separated by centrifugation at 12000*g*, resuspended in SDS-page sample buffer and boiled for 5 minutes before using them for SDS-PAGE analysis.

### RT-PCR Analysis

Total RNA was extracted from cells using Trizol reagent (Invitrogen) according to the manufacturer's instructions and 1μg was used for retrotranscribed (RT) using M-MLV reverse transcriptase (Invitrogen). Five percent of the RT reaction was used as template for PCR analysis (GoTaq, Promega). The sequences of all primers used are listed in Supplemental Table 1.

### SDS- PAGE and Western blot analyses

Protein extracts were separated by SDS-PAGE and analysed by Western blot as previously described [38]. The primary antibodies used are: rabbit anti-actin (1:1000, Sigma-Aldrich); rabbit anti-4E-BP1 (1:1000), rabbit anti-4EBP1 pSer65 (1:500), rabbit anti-eIF4E (1:1000), rabbit anti-eIF4G (1:1000), rabbit anti-AKT pThr308 (1:200), rabbit anti-rpS6 pSer240-244 (1:1000), rabbit anti-rpS6 pSer235-236 (1:1000) (all from Cell Signalling Technology); rabbit anti-pSer473 AKT (1:1000) (from BioSource); rabbit anti-eIF4A (1:1000) (Abcam). Actin was used as loading control to normalize the samples. Secondary IgGs conjugated with horseradish peroxidise (1:10 000; Amersham Bioscience) were incubated for 1 hour and signals were detected by enhanced chemioluminescence (Santa Cruz Biotechnology).

### Colony Formation Assay

Single-cell suspensions were plated in 35mm plates at low density (1000 cells/plate) [22]. After 1 day, cells were treated with inhibitors as indicated in the figures. The medium was changed every 48 hours and inhibitors were added at every change of medium. After 10 days, cells were fixed in methanol for 10 minutes and stained overnight with 5% Giemsa. Plates were then washed twice with PBS and dried. Pictures were acquired using digital camera to count the colonies. Results represent the mean ± s.d. of three experiments.

### Cell Count and viability Assay

Cell count was used to monitor cell proliferation. BON-1 RR and QGP-1 were seeded at 40000 cells/plate in 24-well plates and treated for 72 hours as described. At the end of treatments, cells were washed in PBS, trypsinized and counted using the Thoma's chamber.

For apoptosis assays, cells were seeded at 80 000 cells/plate in 12-well plates and treated with inhibitors as indicated. Cells were fixed for 10 minutes in 4% paraforlmaldeyde (PFA) and permeabilized with 0,1% Triton X-100 for 10 minutes. After 1 hour with PBS with 3% BSA, cells processed for immunofluorescence analysis using the anti-cleaved caspase-3 antibody (1:400 dilution, Sigma-Aldrich) for 1 hour and 30 minutes. Cells were incubated with secondary antibody (1:500 dilution; Jackson ImmunoResearch Laboratories). Five random fields were chosen and at least 100 cells/field were counted. Results represent the mean ± s.d. of three experiment performed in triplicate.

### 7-Methyl-GTP-Sepharose Chromatography

Assembly of eIF4F complex was evaluated essentially as previously described [10, 25]. Briefly, PET cells were resuspended in lysis buffer (see above) containing 0,5% Triton X-100 and 30 U/mL RNasin (Promega). Cell extracts were incubated for 10 minutes on ice and centrifuged at 12000*g* for 10 minutes at 4°C. The supernatant fractions were pre-precleared for 1 hour on Sepharose beads (Sigma-Aldrich) and then centrifuged at 1000*g* for 1 minute. Pre-cleared supernatants were then incubated with 7-Methyl-GTP Sepharose (Amersham Bioscience) for 90 minutes at 4 °C under constant shaking. After three washes in lysis buffer, bound protein were eluted in SDS-PAGE sample buffer and analysed by Western blot

### Protein Synthesis Assay

For the measurement of protein synthesis rate, 2x10^5^ cells were plated in 35 mm dishes in presence of inhibitors as indicated. In the last 30 minutes [^35^S]-cell labelling mix (Perkin Elmer EasyTag ™ Express35S35S, 1000 Ci/nmol) was added to final concentration of 10 ɥCi/mL. Cells were lysed in High Salt Buffer (HSB) (Tris HCl pH 7.5 50 mM, NaCl 350 mM, MgCl2 1 mM, EDTA 0,5 mM, EGTA 0,1 mM) with 1% NP-40) and proteins were precipitated in 10% trichloroacetic acid. After three washes with 5% cold thricloroacetic acid, the insoluble materials was collected on GFC filters (Whatman) and the incorporated radioactivity was measured in scintillation fluid. Results represent mean ± s.d. of three experiments.

## SUPPLEMENTARY INFORMATION TABLE AND FIGURES


