# Supplementary material for: Combined therapy with RAD001 e BEZ235 overcomes resistance of PET immortalized cell lines to mTOR inhibition
Source: Oncotarget. 2014 Jun 18;5(14):5381–91. doi: 10.18632/oncotarget.2111 (PMC4170632; doi:10.18632/oncotarget.2111)
Supplement: Supplementary file 1 [file oncotarget-05-5381-s001.pdf]

Combined therapy with RAD001 e BEZ235 overcomes resistance of PET immortalized cell lines to mTOR inhibition

Supplementary Information

Supplemental Table 1. List of primers used for PCR analyses in this work.

Primer	Sequence
ZEB1 Forward	CATTGCTGACCAGAACAGTGTTCC
ZEB1 Reverse	CGGTCAGCCCTGCAGTCCAAG
ZEB2 Forward	GAGGCGCGCGAGAAAGG
ZEB2 Reverse	GCCCAGCTTCCCGTAGCC
SLUGForward	AGTCCAAGCTTTCAGACCCCCATGCCATTG
SLUG Reverse	TTCTCCCCCGTGTGAGTTCTA
VIMENTIN Forward	AGACACTATTGGCCGCCTGCAGGATG
VIMENTIN Reverse	GAAGAGGCAGAGAAATCCTGCTCTCCTCGCCTTCCA
E-CADHERIN Forward	CACCCCCTGTTGGTGTCTTT
E-CADHERIN Reverse	TGGATCCTCAACTGCATTCCC
ESRP1 Forward	GGCTCGGATGAGAAGGAGTTGAT
ESRP1 Reverse	GAAGGAAGTCCCTACTCCAAT
ESRP2 Forward	ACGCTGCACAAATCGCTGGTT
ESRP2 Reverse	GTGCAGGACCTGTCGCAAT
HPRT Forward	TGACCAGTCAACAGGGGACA
HPRT Reverse	TTCGGGGGTCCTTTTCACC

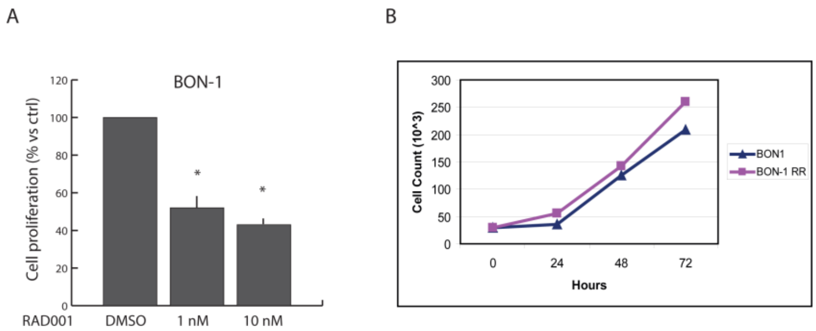

Supplementary Figure 1: Effect of RAD001 on cell proliferation in BON-1 and BON-RR. (A) Proliferation of BON-1 was assayed by MTS assay after 72 hours in presence of different doses of RAD001, as indicated. (B) The graph represents the cell count of BON-1 (without RAD001) and BON-1 RR (in presence of 10 nM RAD001) at different times, as indicated.

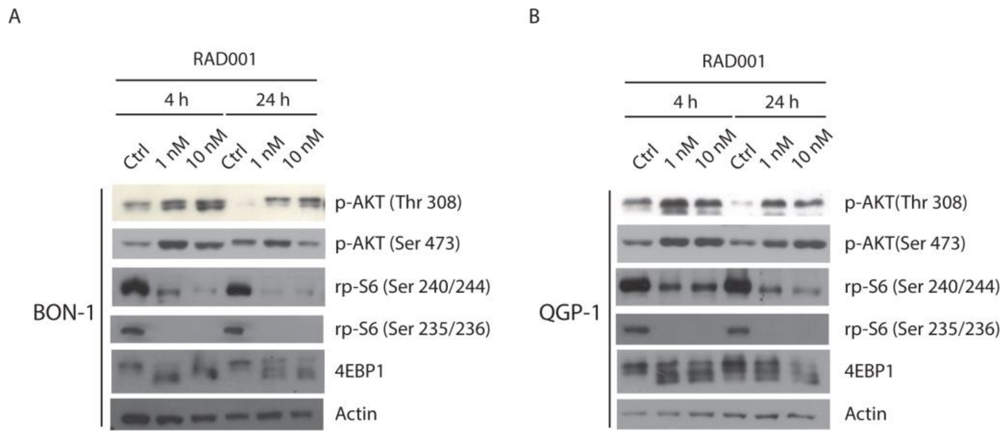

Supplementary Figure 2: RAD001 treatment induces phosphorylation of AKT in PET cell lines. Western Blot analysis of p-AKT in Thr 308 and Ser 473, p-rpS6 in Ser 235-235 and Ser 240-244, and 4EBP1 during treatment with different doses of RAD001 in BON-1 (A) and QGP-1 (B).

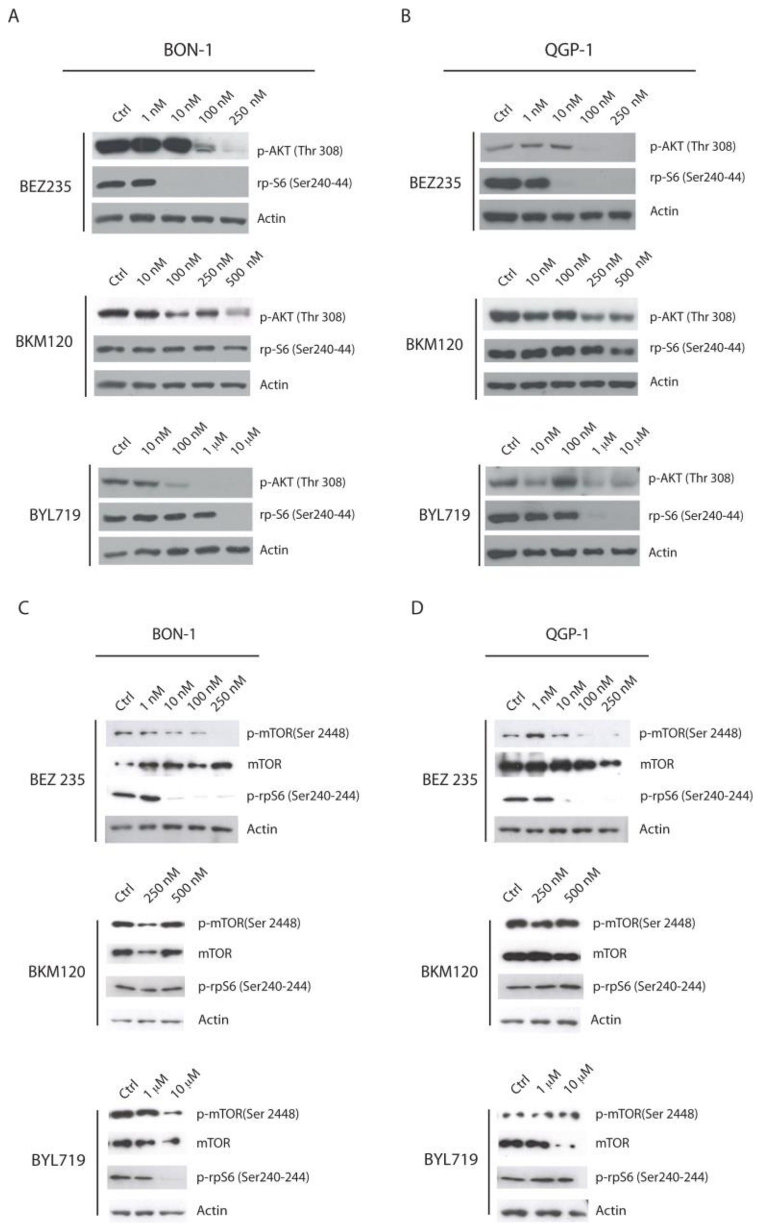

Supplementary Figure 3: Effect of PI3K inhibitors on the PI3K/AKT/mTOR pathway in PET cell lines. Western Blot analysis of p-AKT Thr 308 and p-rpS6 Ser 240-244 in BON-1 (A) and QGP-1 (B) and of mTOR, p-mTOR Ser 2448 and p-rpS6 Ser 240-244 in BON-1 (C) and QGP-1 (D) cells. PET cells were treated for 4 hours with increasing doses of PI3K inhibitors as indicated in the figure.

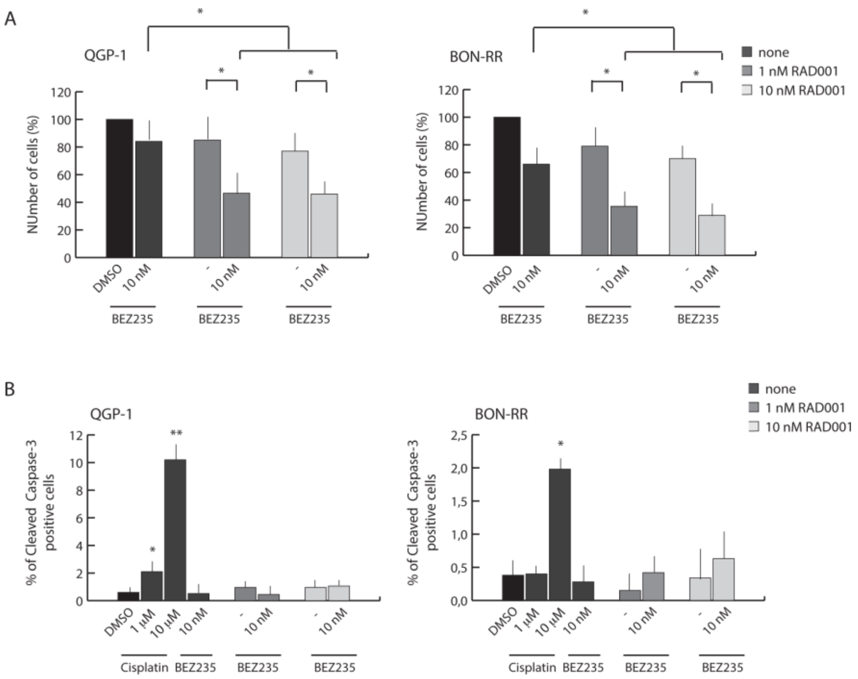

Supplementary Figure 4: Combined treatment with BEZ235 and RAD001 exerts a synergic effect on cell proliferation. (A) Cell proliferation was assayed by cell count after 72 hours in QGP-1 and BON-1 RR cells treated with BEZ235, RAD001 or both inhibitors as indicated. Histograms show the percentage of inhibition of number of cells in comparison to control cells from three experiments (mean ± s.d.). **(B)** Cell death was detected by immunofluorescence analysis of the cleaved form of caspase-3 after 72 hours in QGP-1 and BON-1 RR cells treated with Cisplatin, BEZ235, RAD001 or both inhibitors as indicated. Histograms show the percentage of cleaved caspase-3 positive cells from three experiments (mean ± s.d.). Statistical analysis was performed by the paired Student's t-test; * $P \leq 0.05$, ** $P \leq 0.01$.

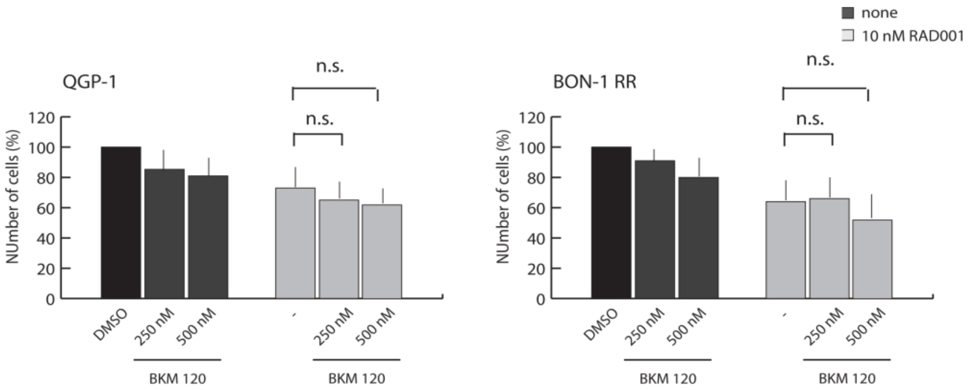

Supplementary Figure 5: Combined treatment with BKM120 and RAD001 does not exert a synergic effect on cell proliferation and cell viability. Cell proliferation was assayed by cell count after 72 hours in QGP-1 and BON-1 RR treated with BKM120, RAD001 or both inhibitors as indicated. Histograms show the percentage of inhibition of number of cells in comparison to control cells from three experiments (mean ± s.d.).Statistical analysis was performed by the paired Student's t-test; * $P \leq 0.05$, ** $P \leq 0.01$.

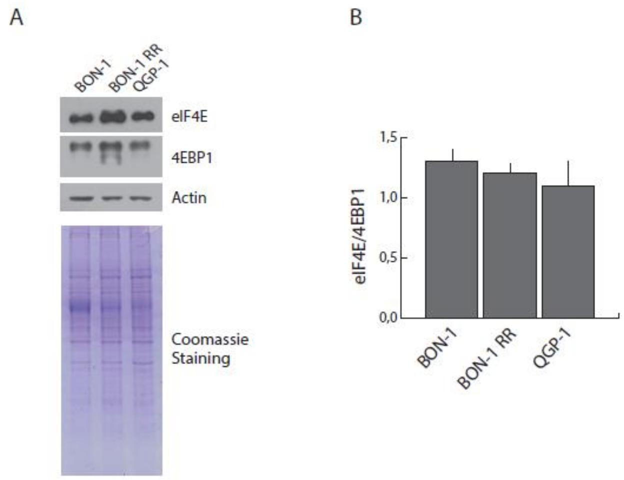

Supplementary Figure 6: RAD001-sensitive and insensitive PET cells display similar eIF4E/4EBP1 ratios. (A) Representative image of Western Blot analysis of eIF4E, 4EBP1 and Actin in PET cell lines. Actin and Coomassie Staining were used as loading control. (B) Densitometric analysis of eIF4E and 4EBP1, after each was normalized with respect to Actin levels. Results are the mean + standard deviation of three independent experiments.
